# Combined Optical Coherence and Fluorescence Microscopy to assess dynamics and specificity of pancreatic beta-cell tracers

**DOI:** 10.1038/srep10385

**Published:** 2015-05-19

**Authors:** Corinne Berclaz, Christophe Pache, Arno Bouwens, Daniel Szlag, Antonio Lopez, Lieke Joosten, Selen Ekim, Maarten Brom, Martin Gotthardt, Anne Grapin-Botton, Theo Lasser

**Affiliations:** 1 Ecole Polytechnique Fédérale de Lausanne, Lausanne, Switzerland; 2 Radboud University Medical Center, Nijmegen, The Netherlands; 3 Danish Stem Cell Center, University of Copenhagen, Copenhagen, Denmark; 4 Institute of Physics, Faculty of Physics, Astronomy and Informatics, Nicolaus Copernicus University, Grudziadzka 5, PL-87-100 Torun, Poland

## Abstract

The identification of a beta-cell tracer is a major quest in diabetes research. However, since MRI, PET and SPECT cannot resolve individual islets, optical techniques are required to assess the specificity of these tracers. We propose to combine Optical Coherence Microscopy (OCM) with fluorescence detection in a single optical platform to facilitate these initial screening steps from cell culture up to living rodents. OCM can image islets and vascularization without any labeling. Thereby, it alleviates the need of both genetically modified mice to detect islets and injection of external dye to reveal vascularization. We characterized Cy5.5-exendin-3, an agonist of glucagon-like peptide 1 receptor (GLP1R), for which other imaging modalities have been used and can serve as a reference. Cultured cells transfected with GLP1R and incubated with Cy5.5-exendin-3 show full tracer internalization. We determined that a dose of 1 μg of Cy5.5-exendin-3 is sufficient to optically detect *in vivo* the tracer in islets with a high specificity. In a next step, time-lapse OCM imaging was used to monitor the rapid and specific tracer accumulation in murine islets and its persistence over hours. This optical platform represents a versatile toolbox for selecting beta-cell specific markers for diabetes research and future clinical diagnosis.

Islets of Langerhans are structures hosting the insulin-producing beta-cells, which play a central role in glucose homeostasis. For a deeper understanding of the pathogenesis of diabetes and for developing beneficial treatments protecting beta-cells, improving their function or promoting their proliferation/regeneration during diabetes, an accurate assessment of the beta-cell volume is necessary. These ambitious goals motivate the search for specific beta-cell markers. The utmost goal is to achieve human *in vivo* imaging of beta cells, which is an on-going worldwide effort of intensive research^1^,^2^.

Non-invasive clinical imaging techniques such as MRI, PET or SPECT rely on contrast agents or radio-ligand tracers to discriminate between the endocrine and exocrine pancreas. However, these clinical imaging modalities cannot provide a sufficient resolution to resolve individual islets, and therefore rely solely on the contrast quality of the used bio-tracer. Therefore, imaging of smaller individual islets requires the higher spatial resolution of optical imaging. Although optical imaging has a limited penetration depth of a few hundred micrometers and therefore would have very limited use in a clinical setting, it can provide an alternative to assess the specificity of beta-cell markers in recognized animal models. In principle, classical optical techniques such as fluorescence microscopy, confocal^3^ or 2-photon microscopy^4^ allow identifying fluorescently labelled beta-cell tracers. However, their voxel by voxel scanning results in long imaging acquisition times, making *in vivo* imaging of the pancreas in the abdominal cavity and time-lapse imaging during the tracer accumulation challenging. Line-scanning confocal fluorescence imaging overcomes this speed limitation^5^ but similarly to other classical optical techniques requires genetically modified mice to visualize pancreatic islets^6^. Optical Coherence Microscopy (OCM) circumvents all these limitations by providing fast, three-dimensional label free imaging of islets of Langerhans^7^,^8^ along with the islet vascularization and blood flow^9^,^10^,^11^.

In this paper, we exploit the advantages of OCM enhanced with a confocal fluorescence channel to assess the specificity and the dynamics of a beta-cell tracer linked to a fluorophore. As a proof of principle, we demonstrate the high beta-cell specificity of a Cy5.5-exendin-3 tracer *in vitro* and *in vivo* for which other imaging modalities have been used and can serve as reference. Exendin-3 is an agonist of the glucagon-like peptide-1 (GLP1) that targets glucagon-like peptide-1 receptor (GLP1R), a promising candidate due to the specificity and the high level of GLP1R expression on beta-cells^12^,^13^,^14^. Using GLP1 agonists, promising results have been obtained with PET and SPECT^15^,^16^,^17^,^18^,^19^,^20^, MRI^21^,^22^ and fluorescence microscopy^3^,^23^,^24^. Our study reveals promising specificity and dynamic features of GLP1 tracers and sets a platform for further characterization of beta-cell tracers.

## Results

### Exendin-3 coupled to Cy5.5 retains efficient binding to GLP1R

**Figure 1:**
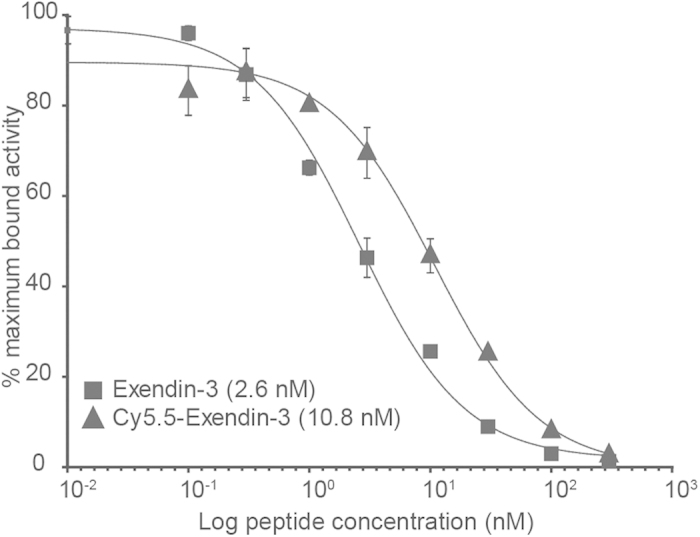
Competition binding assay (IC_50_) of exendin-3 and Cy5.5-exendin-3 on CHL-GLP1R cells. ^111^In-DTPA-exendin-3 was used as tracer. The sample with the highest binding percentage was set at 100%.

The tracer exendin-3 has already been investigated with different modalities^15^,^25^. For an optical monitoring of the exendin-3 binding process to cells expressing GLP1R *in vivo*, we coupled Cy5.5 to exendin-3 to the epsilon amino-group of the C-terminal lysine in analogy to the DTPA conjugated exendin. This strategy allows a straight comparison between the radiolabeled and fluorescently labeled compound. To investigate if this modification alters the pharmacological properties DTPA-exendin-3 was labeled with ^111^InC_3_ with a specific activity of 700 GBq/μmol. The radiochemical purity was 95% as determined by ITLC. Radiolabelled exendin-3 and Cy5.5-exendin-3 were used to determine the IC_50_ diagrams using Chinese hamster lung (CHL) cells stably expressing GLP1R. Both compounds showed a high affinity for the GLP1R. The idem IC_50_ values of exendin-3 and Cy5.5-exendin-3 were 2.6 nM and 10.8 nM respectively (Fig. 1 with 95% confidence intervals of 1.9–3.4 and 7.0–16.7 respectively). These values in the nanomolar range are not significantly different (p = 0.68).

### Cy5.5-exendin-3 binding and internalization *in vitro* in cells expressing GLP1R

**Figure 2:**
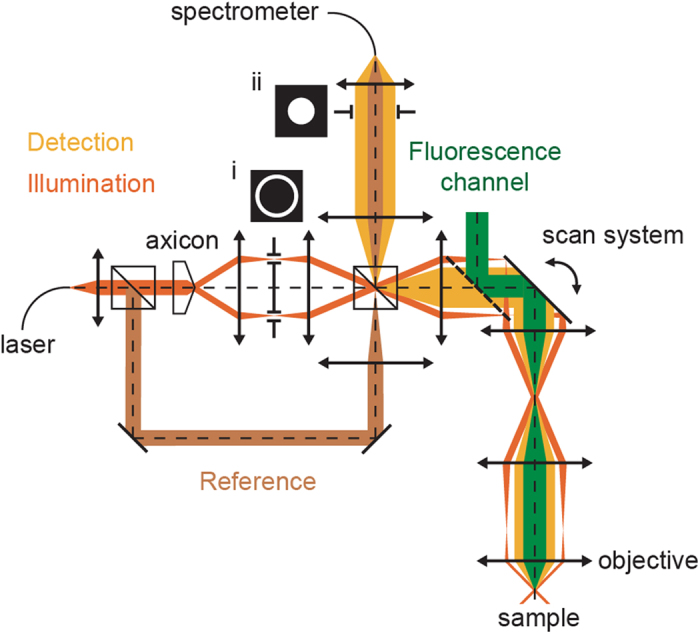
Schematic layout of the dual systems. The dark-field effect is obtained by adding an annular mask (i) in the illumination and a pupil mask (ii) in the detection arm.

To assess the specificity of Cy5.5-exendin-3 for GLP1R, stably transfected CHL cells with the human GLP1R were used and imaged with dark field OCM (dfOCM). The novel instrument we designed (Fig. 2) combines OCM to detect islets or cells based on their natural scattering^8^ with fluorescence to detect the tested tracer. OCM part works in two configurations: extended focus OCM (xfOCM)^26^ and dark field OCM (dfOCM)^27^. xfOCM is optimized for small animal imaging whereas dfOCM possesses dark field contrast enhancement and is designed for cell imaging. dfOCM allows imaging of the weak scattering signal from cells by suppressing the strong reflection originating from the microscope slide.

**Figure 3:**
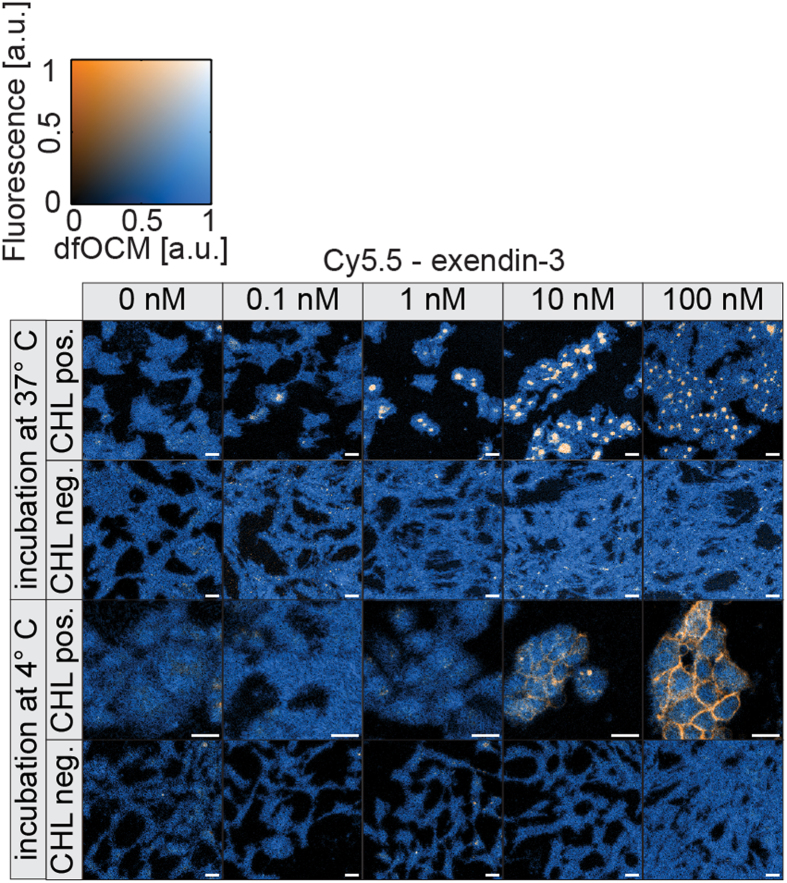
*In vitro* experiments on CHL- hGLP1R cells. Cell imaging with the OCM/fluorescence platform after 90 min of incubation with different concentrations of Cy5.5-exendin-3 (0-100 nM) with CHL positive or negative for hGLP1R. Scale bar: 20 μm.

We investigated a concentration range of 0–100 nM of Cy5.5-exendin-3 on CHL cells positive or negative for GLP1R. To discriminate between internalization and binding, the cells were incubated at either 37 °C or 4 °C for 90 minutes. As shown in Fig. 3, complete internalization is observable down to 1 nM at 37 °C whereas only a membrane staining is seen at 4 °C. In both cases, no signal is detectable in the CHL negative control. As an additional control we used a conventional confocal fluorescence microscope, which confirmed our findings.

### Determination of Cy5.5-exendin-3 dose for a specific islet detection

**Figure 4:**
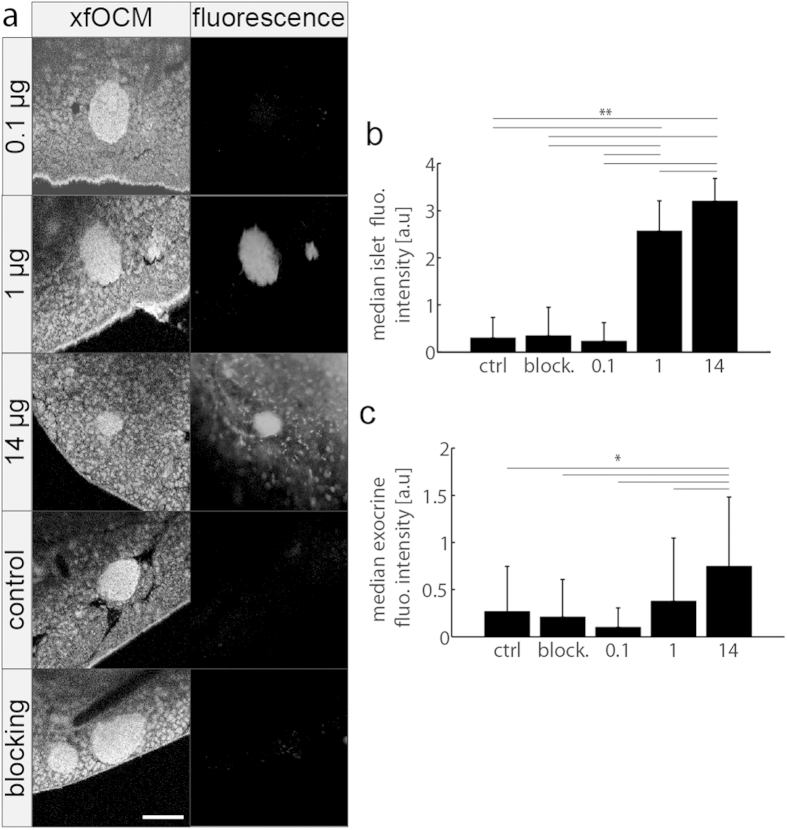
Evaluation of different doses of Cy5.5-exendin-3 in vivo. (a) Representative images of the fluorescence signal and the corresponding xfOCM images 4 hours after injection of different doses of Cy5.5-exendin-3. Scale bar: 200 μm. (**b**,**c**) Quantification of fluorescence islet intensity **(b)** and exocrine fluorescence intensity **(c)** 4 hours after injection of different doses of Cy5.5-exendin-3 (0.1, 1, 14 μg), control (ctrl) and blocking (block.).* p < 10^−4^, ** p < 10^−5^, Mann-Whitney non-parametric U-test.

To determine the minimum dose required for *in vivo* tracer detection of a fluorescence labeled tracer, three different doses of Cy5.5-exendin-3 (0.1, 1 and 14 μg per mouse that is 2.6, 26.3 and 391.7 μg/kg) were investigated. No fluorescent signal inside the islets could be detected 4 hours after the intravenous injection of 0.1 μg of Cy5.5-exendin-3. Only in rare cases (<5%) a weak fluorescence appeared at the limit of observation. Conversely both at 1 μg and 14 μg doses, the tracer accumulates only inside the islet 4 hours after injection (Fig. 4a). Although the fluorescence intensity of islets in mice injected with 14 μg was stronger than at 1 μg of Cy5.5-exendin-3 (Fig. 4b), the background signal in the exocrine pancreas was higher at 14 μg than at 1 μg of Cy5.5-exendin-3 (Fig. 4c), with a dot-like staining in the exocrine pancreas (Fig. 4a). In addition while right after injection of a 14 μg tracer dose, a fluorescent signal appeared in the pancreas vasculature, at an intermediate dose of 1 μg of Cy5.5-exendin-3, no fluorescent signal was observed in the vasculature but a strong fluorescent signal appeared inside the islets right after injection (Fig. S1).

A dose of 1 μg of Cy5.5-exendin-3 is considered to be optimal based on our quantitative analysis. Firstly, the ratio (mean endocrine/mean exocrine fluorescence intensity) was ~6 for a 1 μg dose and decreases to ~4 for a 14 μg dose. Secondly, this finding is supported by the fact that the fluorescence signal with 1 μg dose was well observable at 4 hours (Fig. 4b) after injection and that the signal in the exocrine pancreas was as low as in the control animals (Fig. 4c). In addition, we investigated whether there was a correlation between islets depth localization or its volume. However, no correlation could be found (Fig. S2). No fluorescent response was measured 4 hours after injection of a blocking solution of 100 μg of unlabeled exendin-3 (Fig. 4a,b), confirming the specificity of Cy5.5-exendin-3 for islets of Langerhans.

### Ex vivo organ analysis after *in vivo* imaging reveals the specificity of Cy5.5-exendin-3 for beta-cells

**Figure 5:**
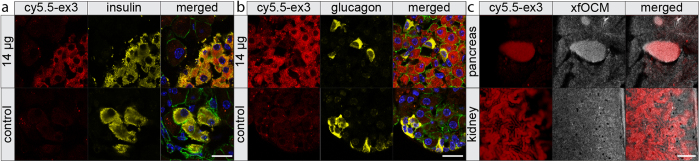
Internalization of Cy5.5-exendin-3. **(a,b)** Representative sections showing the internalization of the tracer specifically in beta-cells **(a)** and not in alpha-cells **(b)**. E-cadherin staining is shown in green. Scale bar: 20 μm. **(c)** Ex vivo signal of the tracer is found in pancreatic islet and in the kidneys. The strong fluorescent intensity in the kidney requires using a pinhole to reject out of focus fluorescence. Scale bar: 200 μm.

To further investigate the specificity of Cy5.5-exendin-3, the pancreata of mice injected with 14 μg of Cy5.5-exendin-3 or only with PBS were sectioned and stained for insulin, glucagon and e-cadherin (Fig. 5a,b) and further analyzed by confocal fluorescence microscopy. This shows that the signal of Cy5.5-exendin-3 is co-localized with the insulin staining and is clearly internalized in beta-cells whereas alpha-cells did not internalize the tracer. No Cy5.5 signal was observed in the control mice injected only with PBS.

We performed an ex vivo study based on fluorescence imaging for establishing the biodistribution in several organs (heart, lung, duodenum, large intestine, stomach, kidney, spleen and liver). After dissection, a specific Cy5.5-fluorescent signal in the islets is still observable (Fig. 5c). Among the organs investigated, only the kidneys showed a strong fluorescent response (Fig. 5c) about 2 times higher than the signal observed in the islets (Fig. S3), which might cause difficulties for PET/SPECT imaging. In our OCM-fluorescence platform the kidneys and the pancreas are clearly distinguishable: no crosstalk was observed during the *in vivo* assessment.

### Cy5.5-exendin-3 targets islets within minutes and is stably detected for hours

**Figure 6:**
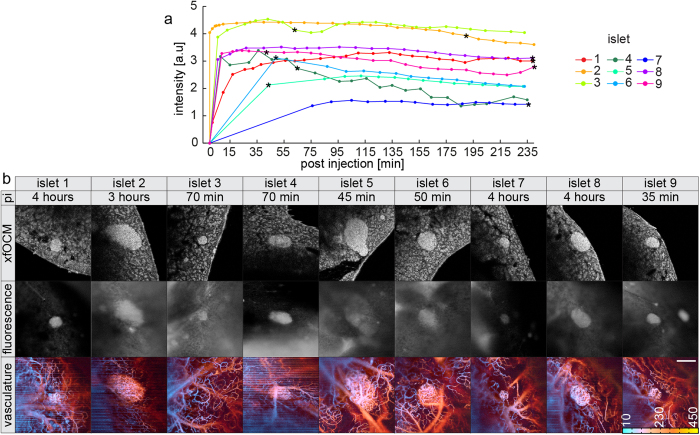
Dynamics of the tracer in vivo. **(a)** Fluorescence signal detected over time after injection of 1 μg of Cy5.5-exendin-3. **(b)** xfOCM (virtual structural section and maximum projection of vascularization) with fluorescence images at different time points indicated with a star symbol in a. Depth is color-coded in micrometers. Stripes in the vascularization images are due to residual pancreas movements. Scale bar: 200 μm.

We monitored the accumulation of the tracer over time in different islets (Fig. 6). No fluorescent signal was detected in islets prior to the ligand injection. Right after injection of the ligand, a signal was visible in the islets and remained stable over 4 hours. Moving red blood cells cause dynamic light scattering, which can be used by OCM for imaging of the pancreas vasculature^28^. This enables a parallel visualization of the tracer, the islet structure and its vasculature with a temporal resolution of ~20 μs per depth-profile. The full 3D volume (512 × 512 × 512 voxel) is imaged in less than 10 seconds for structure and in less than 1 minute for vascularization. The islet vascularization was unaltered after a 4 hour imaging session, which is crucial for a proper delivery of the ligand in the islets. Neither the surgical procedure (the pancreas of mice is exteriorized during the imaging session) nor the imaging is disturbing the blood supply in the islets and in the pancreas.

## Discussion

The identification of beta-cells specific tracers and the characterization of the dose and dynamics of tracer accumulation are mandatory steps on the road towards human noninvasive beta-cell imaging. In this paper, we developed and validated an imaging platform well suited for the task of beta-cell tracer assessment. As a proof of principle we addressed the specificity of exendin-3 by *in vitro* imaging of cells expressing GLP1R and by *in vivo* imaging of pancreatic islets. Using this OCM platform, we demonstrated that: (1) Cy5.5-exendin-3 is internalized *in vitro* by CHL cells expressing GLP1R; (2) *in vivo* for fluorescence labeled tracer investigations a dose as low as 1 μg of the tracer is sufficient to be optically detected and quantified inside islets with a sufficient signal ratio discriminating well endocrine and exocrine tissue; (3) *in vivo* the tracer is specifically internalized as confirmed by blocking experiments with an excess of unlabeled exendin-3; (4) the discrimination of pancreatic islets by the fluorescent tracer is obtained already few minutes after injection and is observable for at least 4 hours. Last but not least, we showed that Cy5.5-exendin-3 is internalized by beta-cells but not by alpha-cells. In contrast to what was suggested by Reiner *et al.*^29^, we show that exendin-3 conjugated with Cy5.5 at the C-terminus lysine in position 40 specifically and efficiently accumulates in CHL cells expressing GLP1R and in the beta-cells *in vivo*.

Compared to classical optical fluorescence microscopy applied to image islets of Langerhans, our OCM platform offers several essential advantages: first, 3-dimensional OCM imaging requires only two lateral scans, resulting in a faster acquisition speed; second, no genetically modified mice to image islets of Langerhans are needed; and third, no injection of an external agent is needed to visualize the pancreas vascularization. However, to access the pancreas, a laparotomy and exteriorization of the pancreas are still required, as it is the case for any optical imaging method. A high beta-cell tracer detection requires good delivery and therefore demands a well perfused organ. The label-free imaging of the vascularization during the whole imaging session proved to be an essential asset for the assessment of tracer dynamics and accumulation. The artifacts visible in some vascularization images in Fig. 6 could be removed by a better stabilization of the pancreas, which remains an experimentally difficult endeavor for small animal imaging. A potential alternative could be the acquisition of additional transversal scan lines to better identify bulk intensity shifts^30^ and to discard them.

To assess the specificity of a potential tracer *in vivo*, optical approaches are needed to resolve individual islets and compare their intensity to other neighboring tissues. While islets can be discriminated due their intrinsic higher scattering signal in OCM, a fluorophore is necessary to detect the tracer. This can sometimes modify the binding properties of tracers, a limitation of the technique also shared by confocal imaging and in general by other techniques offering such resolution in space and time. However, the speed of the technique allows to perform live imaging and quantifications of multiple islets in parallel. Previous assessments of dynamics by confocal microscopy have focused on one islet per mouse, which does not enable to assess the variation in signal^3^. We reveal that there is a range of responses, some islets being 3 fold stronger, but all islets reaching a plateau of signal in less than 15 min. This variability is averaged in PET/SPECT where the signal results from an integrated signal over multiple islets at different depths. The temporal signal over individual islets is however remarkably stable at the plateau, similar to confocal imaging^3^, with little variations due to breathing, heartbeat and peristaltic movements.

Since fluorescence detection is less sensitive than radioactivity detection, we expect differences in the required doses to detect a signal when fluorescence microscopy is used compared to PET/SPECT modalities. Our dose escalation study allows a better understanding of the differences in dosing between fluorescent tracers^3^,^24^ and radio-tracers^15^,^25^. We confirmed that a fluorescence signal is detectable both at 14 μg and 1 μg similarly to other studies based on fluorescence detection of the tracer^3^,^24^. Of note, the 1 μg dose is lower than the doses previously used for confocal microscopy in mouse (2–8 nmol/10–40 μg per animal) but higher than the pharmacologic doses administered in human (5 μg Exenatid per injection). In human imaging, the greater sensitivity of PET will enable to use lower doses a PET/SPECT imaging in mouse only requires around 0.1 μg per animal^16^,^25^. At this dose, no signal is detected in fluorescence, however, similarly to what is observed in PET/SPECT we observed that increased doses result in a decrease of the relative uptake: a 14 μg dose results in a lower target to background when compared to a dose of 1 μg of Cy5.5-exendin-3. It is likely that at 14 μg, we increase the background. In agreement with the biodistribution observed in PET/SPECT a high kidney retention of the tracer was detected.

The presented imaging technology provides a rapid assessment of tracer characteristics *in vivo* complementing the tedious classical ex vivo procedures based on elaborate single time point biodistribution, autoradiography and immunohistochemistry experiments. Our optical imaging platform provides the basis for an efficient and exact *in vivo* dynamic characterization of beta-cell tracer close to the cellular level. Thereby, it offers the possibility to simplify the initial screening to determine whether a potential tracer is specific for beta-cells. Nevertheless, since the tracer detection relies on fluorescence, studies using PET/SPECT to image radio-labeled tracer injected in small rodents are required to determine the suitable dose for clinical imaging. Furthermore, the intrinsic contrast of islets and their vascularization opens the possibility to perform a label-free study during diabetes progression.

In conclusion, this dual-modality imaging allows *in vivo* monitoring of islet structure, vascularization and tracer uptake for pancreas imaging. As shown, our approach is a preclinical and complementary imaging method to MRI/PET/SPECT to characterize beta-cell tracers in mice when the tracer is large enough to be coupled to fluorescent probes.

## Materials and Methods

### Animals and pancreas imaging with xfOCM

All animal experiments were carried out in strict accordance with the recommendations of the federal and local ethical guidelines. The protocols were approved by the local regulatory body of the Canton Vaud, Switzerland (SCAV, authorization 2050). For these investigations, ICR female adult mice were obtained from the Harlan laboratories and underwent laparotomy procedure as previously described^8^. For imaging longer than 30 minutes, the anesthesia was prolonged with 1% isoflurane mixed with oxygen. During the imaging session the mice are kept on a heating stage. The animals were imaged using the xfOCM/confocal fluorescence (Fig. 2) dual system to image the islets (OCM-mode) and the Cy5.5 tracer (fluorescence mode).

### Cell culture and imaging with dfOCM

Chinese hamster lung (CHL) cells stably transfected with the human GLP1-receptor (CHL-hGLP1R)^31^ are a donation from Martin Béhé (PSI, Switzerland). CHL-hGLP1R were grown in Dulbecco’s Modified Eagle’s Medium (DMEM) GlutaMax (Gibco, Invitrogen, catalog 61965) supplemented with 10% heat-inactivated fetal calf serum (vol/vol), 100 units/ml penicillin and 100 μg/ml streptomycin, 50 mg/ml geneticin (G418) sulphate solution (PAA laboratories GmbH, GE Healthcare), 1 mM sodium pyruvate, and 0.1 mM Non-Essential Amino Acids (NEAA), in a humidified 5% CO_2_ atmosphere at 37 °C. hGLP1R negative CHL cells were grown in the same medium but without geneticin. The cells were harvested by trypsinization with trypsin/EDTA. CHL-hGLP1R and CHL negative cells were seeded on μ-Dish 35 mm (Ibidi) and cultured overnight. The cells were washed three times with Krebs buffer (NaCl 7.795 g/L, KCl 0.354 g/L, KH_2_PO_4_ 0.162 g/L, MgS_4_H_2_O 0.293 g/L, CaCl_2_H_2_O 0.374 g/L, NaHCO_3_ 0.424 g/L, Hepes 2.39 g/L) and incubated at 37 °C or 4 °C with Cy5.5-exendin-3 (0–100 nM) for 90 min in Krebs buffer with 3.9 mM glucose. Following incubation, cells were washed three times with Krebs buffer and put at 4 °C before imaging. Cells were imaged using the dfOCM/confocal fluorescence dual system (Fig. 2) to match the signals originating from cells and the Cy5.5-exendin-3 tracer.

### Conjugation of Exendin-3 with fluorophore and radionuclide and IC_50_ determination

^111^InCl_3_ was obtained from Covidien (Petten, The Netherlands) and (DTPA-) exendin-3 was purchased from Peptide Specialty Laboratories (PSL, Heidelberg, Germany). Cy_5.5_ Mono NHS Ester was purchased from Amersham (GE Healthcare, Buckinghamshire, UK) and conjugated to exendin-3 by PSL. Both DTPA and Cy^TM^5.5 Mono NHS Ester were conjugated to the ε-amino group of the Lysine residue at position 40. DTPA-exendin-3 was radiolabeled with ^111^InCl_3_ as described previously^25^.

The 50% inhibitory concentrations (IC_50_) of exendin-3 and Cy5.5-exendin-3 were determined using CHL-GLP1R cells, grown to confluence in 6-wells plates. Concentrations of the unlabeled exendin-3 and Cy5.5-exendin-3 ranging from 0.1 to 300 nmol in DMEM-GlutaMax with 0.5% (w/v) bovine serum albumin (BSA) (n = 3) were added to the cells together with 50,000 cpm ^111^In-DTPA-exendin-3. The cells were incubated for 4 h on ice, washed twice with DMEM-GlutaMax with 0.5% BSA and harvested using 1 ml 0.1 M NaOH. The radioactivity associated with the cells was measured in a well-type gamma counter (Wallac 1480-Wizard, Perkin-Elmer, Boston, MA, USA). The IC_50_ values were calculated by one-site competition analysis with Graphpad Prism (version 5.03, GraphPad Software, San Diego California USA). An unpaired t-test was used for significance determination with a p-value below 0.05 considered as significant.

### *In vivo* evaluation of different doses, blocking experiments, time-lapse imaging and image analysis

Mice were injected intravenously in the tail vein with either 0.1 μg (2.6 μg/kg, 0.5 nmol/kg, n = 4 mice, 65 islets analyzed in total), 1 μg (26.3 μg/kg, 5.5 nmol/kg, n = 5 mice, 68 islets analyzed in total) or 14 μg (391.7 μg/kg, 82.2 nmol/kg, n = 4 mice, 34 islets analyzed in total) of Cy5.5-exendin-3 in 200 μl of PBS and imaged 4 hours after ligand injection. Control mice (n = 5 mice, 24 islets analyzed in total) were injected with PBS only. For blocking experiments, 1 μg of Cy5.5-exendin-3 and 100 μg of exendin-3 (2.8 mg/kg, 0.6 μmol/kg) in 200 μl of PBS were injected intravenously in the tail vein and imaged 4 hours later (n = 4 mice, 56 islets analyzed in total). For time-lapse imaging, mice were injected intravenously with 1 μg of Cy5.5-exendin-3 in 200 μl of PBS and imaged for up to 4 hours (n = 4 mice, 9 islets analyzed in total). Quantification of the fluorescence signal of Cy5.5-exendin-3 was performed on images taken with an open pinhole. The median fluorescent intensity of the pixels belonging to an islet was quantified in a post processing step by a semi-automatic segmentation of the fluorescent signal overlapping the OCM signal of islet. For time-lapse imaging, the time intensity curve was smoothed with an averaging filter of three, i.e. each point represents the averaging of 3 consecutive points.

### OCM instruments for small animal and cell imaging

**xfOCM**  All *in vivo* imaging was performed with our xfOCM instrument^11^,^26^ equipped with a Zeiss Neofluar objective (10x; NA 0.3, Carl Zeiss) with a lateral resolution of 1.3 μm and a depth of field of ~400 μm. The illumination power on the pancreas was around 5 mW to acquire a full profile over the extended depth. Imaging the pancreas vascularization is based on a specific scanning protocol^32^ and a phase variance algorithm^10^. This method uses a specific scanning protocol where each line (B-scan) is scanned several times. For our measurements, 8 B-scans were taken at 50 kHz (18 μs integration time per depth-profile). The circular variance of temporal phase changes was calculated to extract the vascularization. To improve the contrast, the circular variance was averaged over a window of 8 pixels in the axial direction.

**dfOCM**  The dark field OCM (dfOCM)^27^ is used for cell imaging. dfOCM suppresses all specular reflections which originate from the sample slide. For *in vitro* cell imaging, the added darkfield mask placed in a conjugated plane to the back focal plane of the objective increases the contrast for cell measurements substantially. Our dfOCM instrument contains a plan apochromat immersion objective (25x; NA = 0.8, Carl Zeiss) resulting in a 900 nm lateral resolution and an the work isotropic is not correctly used here since we have a lateral resolution of 900 nm and an axial resolution of 3 μm. It is therefore better to remove it. 3 μm axial resolution over a field depth of 50 μm.

Both instruments have a fluorescence channel using the same scanning unit for a simultaneous acquisition of the fluorescence and the OCM signal. For suppressing any crosstalk between the OCM and fluorescence channel detection, a high pass filter (HQ720lp, Chroma Technology Corp.) is placed in the illumination arm. For the fluorescence excitation, a tunable supercontinuum laser was used (Koheras SuperK Extreme, NKT Photonics). A dichroic mirror (720dcxr, Chroma Technology Corp. and a KG1 Schott infrared absorber) in combination with a complementary excitation filter (HQ680/35m, Chroma Technology Corp.) rejects the NIR spectrum and enhances the SNR for the fluorescence detection. Excitation and emission light was separated by a dichroic mirror and excitation filter (z647RDC, Chroma Technology Corp. and Z635/10x, Chroma Technology Corp.). The fluorescence signal was detected by an avalanche photodiode (SPCM-AQR-14-FC; PerkinElmer) and digitized with a NIDAQ card (National instruments).

### Immunofluorescence

At the end of the imaging session, mice were intraperitoneally injected with 1 ml/kg body weight of a solution of pentobarbital (150 mg/ml). The deeply anesthetized animals were transcardially perfused for 2 minutes with PBS and for 8 minutes with 4% paraformaldehyde (PFA) solution. The pancreas was further fixed for 1 day at 4 °C in 4% PFA, prior to an overnight incubation in a 30% (wt/vol.) sucrose solution in PBS at 4 °C. The pancreas was embedded in Optimal Cutting Temperature compound and frozen in isopentane cooled with dry ice. 8 μm cryosections were prepared for staining. The sections were permeabilized 10 minutes with PBS-TritonX100 0.25% and blocked in 10% fetal bovine serum for 30 minutes at room temperature. Incubation with primary antibodies (guinea pig insulin 1:50 Dako, rabbit glucagon 1:200 Cell Signaling, rat e-cadherin 1:100 Takara Bio) was performed at 4  °C overnight in a humid chamber. Secondary antibodies were incubated 45 minutes at room temperature. The sections were further stained with Dapi (1:10000) for 10 minutes at room temperature and mounted with DABCO mounting medium. These immunostained samples were inspected with a ZEISS LSM 710 microscope equipped with a 63x oil Plan-Apochromat objective (NA = 1.4).

### Statistical analysis

Data are presented as mean with standard deviation.

## Additional Information

**How to cite this article**: Berclaz, C. *et al.* Combined Optical Coherence and Fluorescence Microscopy to assess dynamics and specificity of pancreatic beta-cell tracers. *Sci. Rep.* **5**, 10385; doi: 10.1038/srep10385 (2015).

## Supplementary Material

Supporting Information
