# Supplementary material for: Combined Optical Coherence and Fluorescence Microscopy to assess dynamics and specificity of pancreatic beta-cell tracers
Source: Sci Rep. 2015 May 19;5:10385. doi: 10.1038/srep10385 (PMC4437378; doi:10.1038/srep10385)
Supplement: Supporting Information [file srep10385-s1.pdf]

Supplementary Information

Combined Optical Coherence and Fluorescence Microscopy to assess dynamics and specificity of pancreatic beta-cell tracers

Corinne Berclaz[1*], Christophe Pache[1], Arno Bouwens[1], Daniel Szlag[1, 4], Antonio Lopez[1], Lieke Joosten[2], Selen Ekim[2], Maarten Brom[2], Martin Gotthardt[2], Anne Grapin-Botton[3], and Theo Lasser[1]

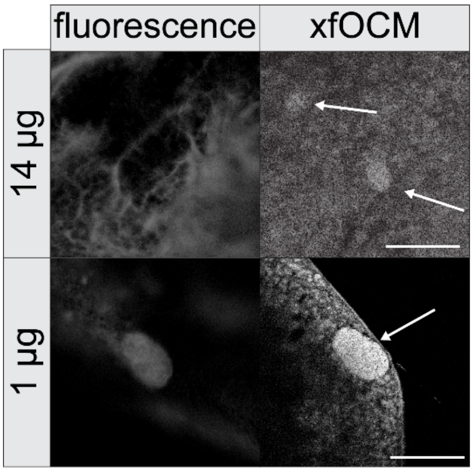

S1 Fig. : Evaluation of different doses of Cy5.5-exendin-3 in vivo. Fluorescence and xfOCM corresponding image a few minutes after injection of 14 µg or 1 µg of Cy.5.5-exendin-3. After injection of 14 µg, the tracer is visible in the vasculature of the pancreas. Arrows indicate islets in the xfOCM image. Scale bar: 200 µm.

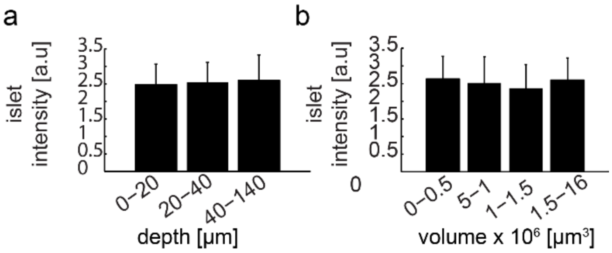

S2 Fig. : In vivo accumulation of Cy5.5-exendin-3. Median fluorescence intensity of the islets 4 hours after injection of 1 µg of Cy5.5-exendin-3 with respect to the islet depth position in the tissue (a) or to the islet volume (b).

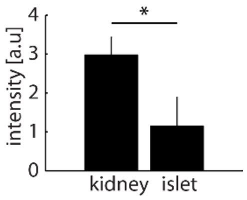

S3 Fig.: Ex vivo quantification. Median fluorescent intensity of the islet and kidney ex vivo 4 hours after injection of 1 µg of Cy5.5-exendin-3. $p<0.01$ with a Mann-Whitney non-parametric U-test.
